# Snake-shaped tumor protruding from an ureteral orifice: a case of a long ureteral fibroepithelial polyp successfully resected ureteroscopically

**DOI:** 10.1093/jscr/rjaf199

**Published:** 2025-04-11

**Authors:** Junya Kawabata, Hideki Takeshita, Yohei Okada, Wataru Hirata, Ayano Ishida, Kojiro Tachibana, Shoichi Nagamoto, Sachi Kitayama, Akihiro Yano, Satoru Kawakami

**Affiliations:** Department of Urology, Saitama Medical Center, Saitama Medical University, Kawagoe, Japan; Department of Urology, Saitama Medical Center, Saitama Medical University, Kawagoe, Japan; Department of Urology, Saitama Medical Center, Saitama Medical University, Kawagoe, Japan; Department of Urology, Saitama Medical Center, Saitama Medical University, Kawagoe, Japan; Department of Urology, Saitama Medical Center, Saitama Medical University, Kawagoe, Japan; Department of Urology, Saitama Medical Center, Saitama Medical University, Kawagoe, Japan; Department of Urology, Saitama Medical Center, Saitama Medical University, Kawagoe, Japan; Department of Urology, Saitama Medical Center, Saitama Medical University, Kawagoe, Japan; Department of Urology, Saitama Medical Center, Saitama Medical University, Kawagoe, Japan; Department of Urology, Saitama Medical Center, Saitama Medical University, Kawagoe, Japan

**Keywords:** fibroepithelial polyp, benign ureteral tumor, ureteroscopy, ureteroscopic resection, minimally invasive

## Abstract

A fibroepithelial polyp (FEP) is a rare benign ureteral tumor that causes gross hematuria and ureteral obstruction. Diagnosis of FEP is difficult using ultrasound or radiographic imaging, and ureteroscopic diagnosis through direct vision is important. Although ureteroscopic resection is less invasive, FEP sometimes grows significantly in both length and size and requires open or laparoscopic ureteral resection. Here, we report a case in which we diagnosed and resected a 9.5 cm long FEP, originating from the mid-ureter, ureteroscopically. A 32-year-old woman with gross hematuria was referred to our hospital. Cystoscopy revealed a snakelike tumor protruding from the left ureteral orifice into the bladder. Ureteroscopy revealed a smooth tumor surface, with the root sufficiently narrow for resection. Based on these features, the tumor was diagnosed as an FEP and was ureteroscopically resected without postoperative ureteral strictures. Ureteroscopy is essential for the diagnosis and treatment of a long FEP.

## Introduction

Most primary ureteral tumors are malignant, whereas benign tumors are rare. Fibroepithelial polyps (FEP) are rare benign tumors of mesodermal origin that occur in the ureter and ureteropelvic junction [1–3]. A total of 87% of FEP cases occur as solitary lesions but sometimes occur as multiple lesions. The median size of the polyps was 4 cm [1]. If the FEP grows significantly in both length and size, it may cause hematuria, ureteral obstruction, and hydronephrosis [1, 4]. Its diagnosis is relatively difficult, and one of its characteristics is that it is sometimes difficult to distinguish it from ureteral cancer. The first choice of treatment for FEP is ureteroscopic resection; however, if the FEP is large or occurs as multiple lesions, this may be difficult. Therefore, other methods, such as laparoscopic surgery or laparotomy may be used [1, 3, 5].

In this case, we encountered a long FEP growth that protruded like a snake inside the bladder. Although we were initially confused about the diagnosis because of its striking appearance, we were eventually able to safely diagnose and treat the condition using ureteroscopy. Below are the details of the diagnostic and treatment courses that we encountered.

## Case report

**Figure 1:**
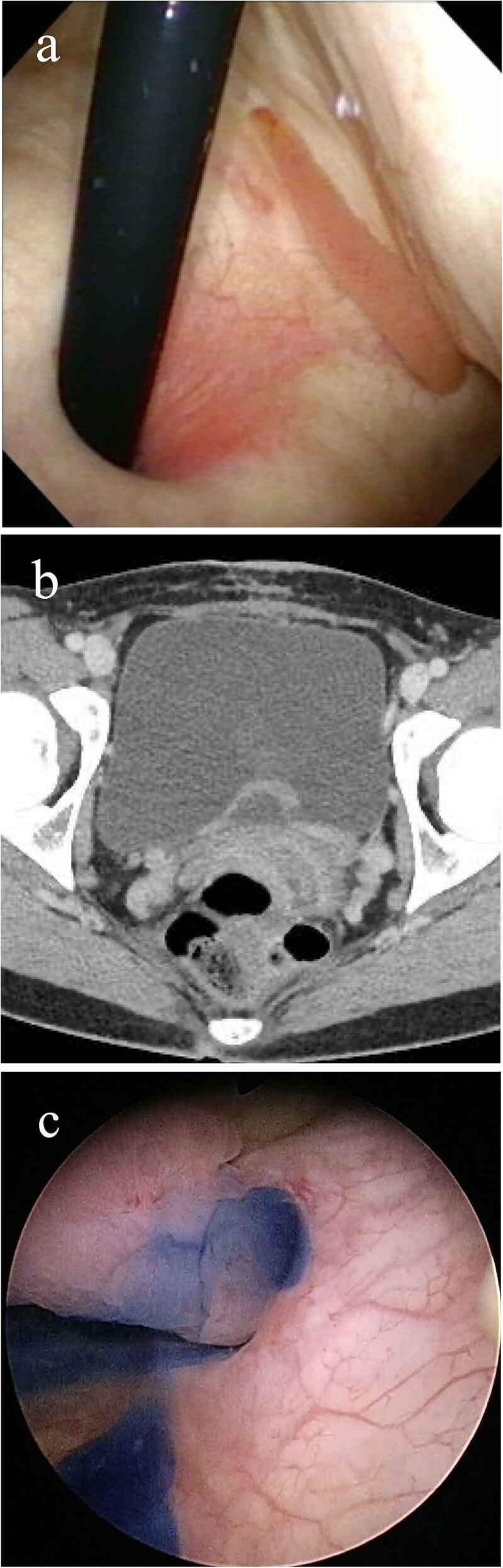
Imaging study findings. (a) Cystoscopy finding. Cystoscopy revealed that a tumor protruded from the left ureteral orifice into the bladder lumen, showing serpentine locomotion with respiratory movements. (b) Contrast enhanced computed tomography finding. Soft tissue shadow was detected around the left ureteral orifice, revealing a long thin tumor. (c) Indigo carmine was excreted through the left orifice that is occupied by the tumor.

A 32-year-old Japanese woman presented to our hospital with painless intermittent gross hematuria that had persisted for 5 months. She had no history of urolithiasis or urinary tract infections. Her blood test results were within normal limits. Although urine analysis revealed microscopic hematuria, urine culture, and cytology tests were negative. Cystoscopy performed in the outpatient clinic revealed a smooth-surfaced snake-like tumor protruding from the left ureteral orifice into the bladder lumen, moving like snake’s crawling with respiratory movements (Fig. 1a). Contrast-enhanced computed tomography (CT) revealed a 3-cm-long soft tissue shadow around the left ureteral orifice (Fig. 1b). Urolithiasis or hydronephrosis was not observed.

**Figure 2:**
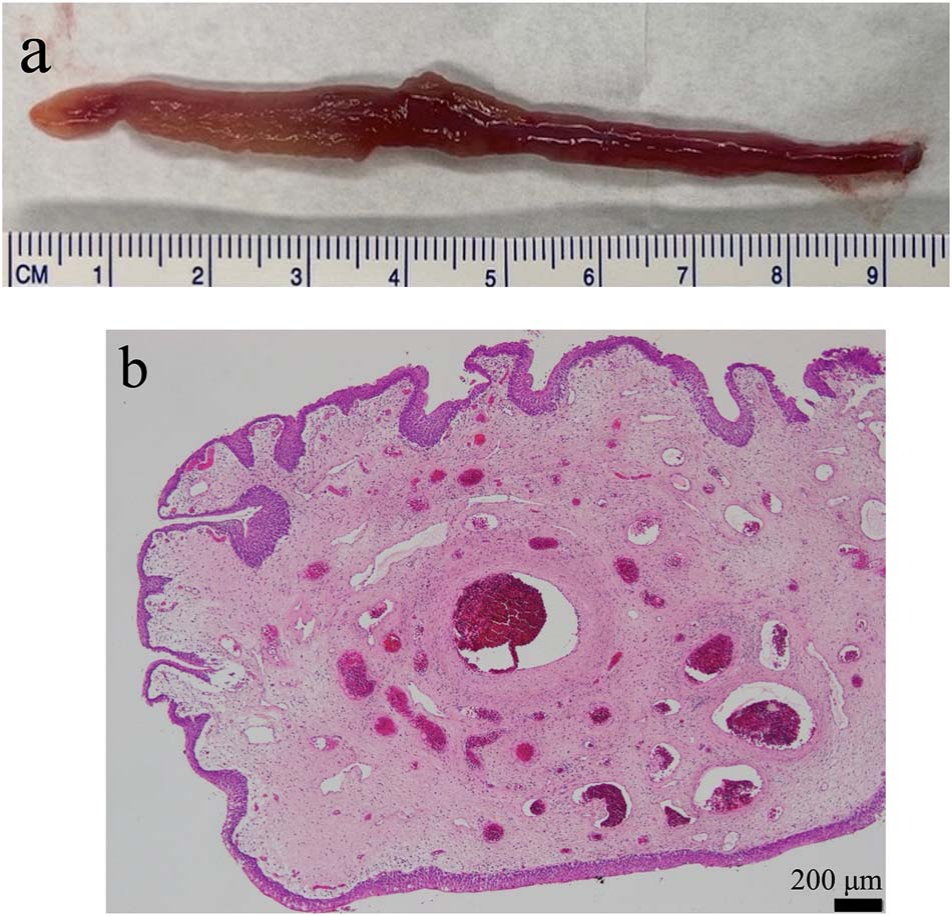
Findings from the excised specimen. (a) Macroscopic finding. The length of the tumor was 9.5 cm. (b) Microscopic finding (×10). A polyp with edematous stroma. The surface layer of the polyp was coated with atypical urothelial cells.

The patient underwent detailed endoscopy in the operating room (Video 1). The indigo carmine test revealed patency in the ureteral orifice occupied by the tumor (Fig. 1c). A ureteroscope was inserted along the tumor into the left ureter. The lower ureter was occupied nearly completely by the tumor, but the ureteroscope could pass through the small gap between the tumor and the ureteral wall. As the scope reached the mid-ureter, the root of the tumor eventually became thinner, and a single narrow base of the tumor was identified. FEP was diagnosed based on the characteristic surface findings and root shape of the tumor. Because this polyp could be safely removed ureteroscopically, ureteroscopic resection was performed using a Holmium: YAG Laser (0.3 J, 10 Hz). Electrocautery was applied to the base of the polyp, and the coagulation area was kept as small as possible. Finally, the polyp was resected and extracted entirely from the ureter and into the bladder lumen using forceps. A double-J stent was inserted, and the ureteroscopic procedure was completed. The stent was removed a month after the surgery. Her gross hematuria stopped, and she reported no complications. Histopathological examination revealed a 9.5 cm tumor and a polyp with edematous stroma, which was confirmed to be ureteral FEP (Fig. 2a and b).

## Discussion

FEP is considered a relatively rare disease. This is because half of the patients diagnosed with FEPs are asymptomatic hence diagnosed incidentally [6]. FEPs can cause gross hematuria, which can lead to misdiagnosis of upper tract urothelial carcinoma [7]. FEPs are difficult to diagnose because their clinical and imaging findings are non-specific. The sensitivity of imaging was reported to be 27% for intravenous pyelography and magnetic resonance imaging, 50% for contrast enhanced CT, and 61% for ultrasonography performed by a skilled examiner [5]. However, with technical advancements in ureteroscopy, the number of reports of FEPs treated with ureteroscopy has gradually increased over the past 20 years [4, 8]. As mentioned in the current case, identifying the characteristic smooth surface of FEP is important for diagnosis because of the low sensitivity of imaging tests [3, 9].

Previously, FEP was treated with open surgery, including ureterotomy, partial ureterectomy, and nephroureterectomy [1, 10]. Before 1985, treatment of FEP ureteroscopically was performed in 0%, but this increased to 68% recently from 2005 to 2014, with the wide spread availability of ureteroscopes [1]. However, open or laparoscopic surgery may still be required. The treatment varies depending on the size, location, and number of polyps [7]. If the tumor is significantly large or tall, proximal, or polypoid, ureteroscopic resection is less likely chosen [1]. Recently, robot-assisted surgery has been reported [2].

Similar to this case, there have been some reports of FEP protruding into the bladder [4, 7, 8]. In such cases, staining urine with intravenous indigo carmine is useful for checking ureter patency and determining whether ureteroscopic management is feasible [11]. If the ureteroscope is successfully inserted and the root of the tumor is observable, thin, and single, ureteroscopic resection is considered. When the polyps are significantly large or multiple, they occupy the lumen of the ureter and are difficult to distinguish from the ureteral wall, making visualization poor [1, 3, 5]. Ureteral stricture is the most common complication of ureteroscopy [10]. Ureteral strictures occurred in 1.8% of patients who underwent treatment of FEP ureteroscopically [1]. Therefore, if the tumor root is significantly large and extends almost halfway through the ureteral wall, treatment options other than ureteroscopy should be considered to avoid postoperative ureteral strictures.

In conclusion, long tumors protruding from the ureter into the bladder may be FEPs. Detailed direct observation of the tumor surface and base using ureteroscopy allows for the diagnosis of FEP. It also helps determine whether ureteroscopic resection is safe by visualizing the relationship between the tumor and ureteral wall. Ureteroscopy is the best choice for diagnosis and selection of a definitive surgical procedure.

## Supplementary Material

FEPvideo2_rjaf199
